# Aristotle and Theophrastus

**Published:** 1851-06

**Authors:** 


Art. II.—*Aristotle and Theophrastus.* Translated from the French of M. Cap. By W. H. Cobb, M.D., of Louisville, Ky.

Aristotle was born at Stagira, about the end of the fourth century before Christ. His father, Nicomachus, was physician to Amyntas, father of Philip, King of Macedon, and claimed to be descended from Machaon, son of Æsculapius. Left an orphan at a tender age, Aristotle was raised by his teacher, Proxenus. His education was conducted with zeal and ability, which, however, did not hinder the young pupil from rapidly squandering his patrimony. He then became a soldier, and returned to Athens, where, deprived of his resources, he betook himself to selling medicine about the public places, and thus reaped the advantages of the medical knowledge acquired from his father and preceptor. Truly it is not for us, as did Epicurus, to reproach Aristotle with having followed the occupation of an apothecary or root-gatherer. Theophrastus remarks that a large number of physicians and estimable men devoted themselves to the study of plants, and drew thence many useful medicinal agents. Who knows that it were not, while engaged in these researches, that Aristotle acquired the taste, and later the genius for natural history?

It was at this same epoch that Plato opened at Athens his celebrated school of philosophy. From that time and during the space of almost twenty years, Aristotle became one of his most assiduous pupils. He himself opened a school, in which he contended with Socrates for the palm as a philosopher and an orator. Already had he devoted himself to philosophy, eloquence, and natural history, and his reputation had attained such a pitch that Philip of Macedon, a short time after the birth of Alexander, wrote to him the following celebrated letter: "Philip, King of

Macedon, to Aristotle, greeting: Know that there is born unto me a son; I thank the gods, not so much for having given him unto me, as for having brought him into this world during the time of Aristotle. I hope you will make him worthy to succeed me and to command the Macedonians." Some time after the death of Plato, the Athenians, having declared war against Philip, he betook himself to Atarmé, of which his friend Hermeas was governor. When his friend perished by the treason and cruelty of Artaxerxes, the philosopher immortalized his memory by a beautiful piece of poetry, raised for him a tomb, and espoused Pythais, his sister. About the same period, Philip called him to his court and entrusted him with the education of Alexander, then thirteen years of age.

The master and pupil passed together many years, in their retreat in a country named Nympheum, near Miéza. Philosophy, belle lettres, and the arts, the physical and natural sciences, and even medicine entered into the plan of his education. When Philip had perished by the knife of the assassin (336 years before Christ), Aristotle remained at court up to the time, when Alexander carried the war into Asia. By many it is pretended that he accompanied him in his first expeditions, but he hastened to return to Athens, where he instituted the Lyceum, and founded his new school of philosophy. After the death of Alexander, Aristotle was attacked by certain rival philosophers and fanatics, who accused him of impiety. He imagined that he ought not to maintain the controversy, and to save the Athenians, as he said, a new attack upon philosophy, he retired to the isle of Eubea, where he died at the age of 63 years. The variety, or rather the universality, of knowledge which characterises the writings of Aristotle, renders peculiarly difficult the task of presenting even a simple analysis of the works of this eminent man. Nevertheless, as we are to consider him merely with a view to the impulse which he was known to have imparted to physics and natural history, we will endeavor to confine ourselves to the limits which our subject prescribes.

Physics was, at this time, an inherent constituent of philosophy, of which it formed one of the principal branches. Aristotle, a pupil of Plato, adopted for a long time the doctrines of the Platonic school; but his more methodical and less impulsive nature was always satisfied with hypotheses, even of the most ingenious character, when not founded upon the reality of the phenomena presented; he perceived the necessity of opening for himself a new path, by giving to science a more solid groundwork, and he did not hesitate to forsake the doctrine of his master and to profit by his errors.

The philosophy of Plato evidently resembles that of Pythagoras. His system, that of skepticism, rests upon doubt of everything in nature. Aristotle, on the contrary, establishes as a rule that all knowledge acquired by our senses when properly directed, should be regarded as certain, and that nothing can be taken cognizance of by the mind, except through the medium of the senses. Plato and Aristotle, we see, are the chief of the two great parties which have divided philosophers from antiquity up to the present period. The one attributed to our ideas an existence independent of the testimony of our senses, and pretended to determine from the definition of things as to their real nature; the other affirmed that our ideas have their origin in observation and experience, and are only born or develope themselves by abstraction. In these two rival camps, are ranged successively the philisophers of all ages. With the followers of Plato, the idealists and realists; with the peripatetics, the nominalists and empirics. In our days the ideologists as yet cling to the system of Plato, while those who cultivate the physical sciences acknowledge that they are indebted to the system of Aristotle for all they know positively about things in nature. In this point of view, Lock and Newton may be regarded as the chiefs of the modern school of peripatetics. The Italian school attributed to the elementary atoms, a definite form, which however did not rest upon a real base. Aristotle did not acknowledge the existence of this hypothetical figure thus given to the primitive particles of matter. While he admitted the four elements of Empedocles and Hippocrates, he added to them a fifth, which he called Ether, an immaterial principle, which he supposed to be perpetually in motion around the earth, considered as a common centre. This is the first idea of the system of whirlwinds. Like Empedocles, he resolved bodies into a number of elements; but he believed that even while in a state of combination, they possessed the original properties of the principles composing them. Finally, he attributed to the influence of the earth, as the heaviest of the elements, the tendency recognized in bodies to move towards a common centre, and to fire as the lightest, and in view of its radiation from the centre towards the circumference, all tendency contrary to that which we term gravity. Do we not perceive even here the germ of the theory of gravitation, and of the expansive power of heat?

Hippocrates had demonstrated that experience is the only means of arriving at, in science, certain results. Upon this principle Aristotle laid the foundation of his new philososhy ; but, to make an application of it, he must discover a surer route than that trodden by the philosophers who preceded him. See by what a train of reasoning he attained this route. The soul acquires no knowledge without the intervention of the senses, whose office it is to put in communication with external objects. From the facts thus acquired, are framed the opinions which constitute the sciences. We must then commence by observing bodies in nature, and the phenomena appertaining to them; but the senses being subject to error, the mind has need of rules to govern it in the midst of the impressions which it perceives; hence the necessity of subjecting the senses to a proper method of observation, and of directing the mind to those processes of reasoning which render error impossible. This last operation constitutes a branch entirely different from philosophy, of which Aristotle may be considered the founder and which bears the name of Logic. These starting points once established, the philosopher of Stagira conceived the project of constructing upon a new plan, the entire edifice of science. The basis of this edifice should be the history of nature. The first thing to be accomplished, was to assemble the material for the researches which belong to it; before the time of Aristotle these materials were but few. The preceptor of Alexander taught his pupil that the most brilliant trophies of his conquest would be to collect the natural productions of the countries, into which he carried his arms, to subserve the progress of natural history. His glorious expedition having opened to the Greeks the gates of India, Persia and Egypt, and having multiplied their relations with the East, the young conqueror collected at a great expense, throughout all the countries he had subdued, the natural productions, but more especially the animals which inhabit them, and sent them to Aristotle. According to the testimony of Pliny, many thousands of persons were charged with collecting these objects, and we are assured that enormous sums (about three millions of our currency) were expended upon them. Thus Aristotle found himself placed in the most favorable circumstances for enriching natural history by a crowd of discoveries.

The history of animals is that branch of natural history which owes most to the works of Aristotle, that which he created, so to speak, and to which he devoted himself with the most perseverance and success. Before him, this science did not exist. In ancient Greece, zoology was less a subject of study for philosophers than for artists, who in it sought for symbols and emblems, and represented certain divinities under the figure of animals consecrated to them. Democritus, Empedocles and certain other anatomists had studied a small number of beings, in an isolated point of view, but they had considered their nature merely in a practical manner, whereas Aristotle laid the foundation of a general and complete science. Not only did he penetrate into the details of zoology but he considered it as a whole, in an elevated point of view, and made it rather the history of the animal kingdom than that of an individual species; he classified the facts as observed, following the different organs and their functions, and by comparing them one with another, he created the science of comparative anatomy. Besides the regular researches and numerous observations peculiar to himself, he applied himself to refuting a crowd of prejudices relative to natural history; by this, I do not mean that he never committed any error himself, but that it should induce us the more readily to pardon them in him, although he has sometimes been reproached with a want of method, yet it is certain that he is the first who introduced order and shed light abroad among the numerous objects which he studied. According to Cuvier, the principal divisions which he established in the animal kingdom are recognized even in our day, and he has pointed out a number to which we have lately retnrned, after having very improperly discarded them. Let us add to this homage, rendered to Aristotle by one of our greatest naturalists, that of Buffon, "I do not believe,' says he, 'it would be possible to reduce to a smaller number of terms, all we have to say upon this subject." It required a genius like his, to preserve as much order and perspicuity. This work appears to my eyes as a table of information collected, with the greatest care, from many thousands of works filled with descriptions and observations of every variety. It is the most scientific abridgement ever yet made, and even when we take it for granted that Aristotle borrowed from the books of his time what he put in his own, the plan of the work, his distribution, the choice of the examples, the justness of his comparisons, a certain turn in his ideas, which I should call the philosophical character, do not permit us to doubt for a moment that he himself was far richer than those from whom he borrowed.

Aristotle had written upon plants, two works entitled the "Theory of vegetables," which have not descended to us. The study of plants having been the object of his first labors, it is evident he had no opportunity of making, in the vegetable kingdom, the useful discoveries which his pupil Theophrastus afterwards collected. Linnæus has considered him a botanist, in dedicating to him, under the name of Aristotelia, an elegant shrub growing in Chiil. Aristotle was impressed with the idea that the whole race of natural beings constitutes an uninterupted line, progressive from the simple to the compound, and even that this series passes from inanimate beings to vegetables and animals. He compared with plants certain marine animals, and insects, from which it is possible to remove many members without rendering life extinct. He thought, according to the doctrines of the day, that vegetables were warm and dry because they are born of the earth and the sun is their father; it is for this reason, says he, that the most fragrant flowers and savory fruits are produced by the richest soils, and those most exposed to the burning sun.

This philosopher bestowed much study upon the writings of Hippocrates, and in medicine possessed a vast amount of knowledge, Diogenes Lærtius attributes to him many works on therapeutics which are lost at the present day. Many good precepts of practice are scattered here and there throughout his writings; he has also given much useful information upon the uses of medicines.

But however comprehensive the history of nature may be, this undertaking could not satisfy a genius so ardent, so universal, so indefatigable as that of Aristotle. By turn, meditating upon each branch of human knowledge, he seemed to embrace the whole of them in his vast mind. Emerging from the depths of philosophy and metaphysics, he enters upon physics properly so called, zoology, botany and medicine. To him was reserved the honor of shedding light upon a crowd of points entirely unknown to his first studies, and of imposing upon human reason, as upon the sciences, belles-lettres and the arts, a code of precepts eternal and imperishable. Thus it is he wrote upon politics, from which he deduced general principles, by comparing the one with the other, the constitutions of all the governments known at that epoch. In his work on poetry, he, for the first time, established the theory of the arts, as reducible to the single principle, the imitation of nature, and fixed the true rule of taste, from the writings of Homer, the chefs d'œuvres of tragedy among the Greeks, and from the best works of cotemporary poets. His rhetoric contains (upon every species of literature,) the most luminous views, the truest ideas, and there it is we find the germ of all, that has ever since been written upon belles-lettres and the arts. In his logic, he directs the march, the advance of reasoning, marks out for it a sure path, and pursues sophistry even in its most specious windings. His moral philosophy presents an analysis full of subtlety and of sagacity, of all the thoughts peculiar to the human mind, every virtue, every vice; in a word, in the sciences he amassed an incalculable treasure of facts and observations, laid the foundation of the animal kingdom and comparative anatomy, and extended and perfected physics; in philosophy he created logic, proclaimed the principle of human knowledge, established the theory of the arts, shed light upon politics and moral philosophy, and everywhere imposed principles which will ever be considered as one of the most brilliant results of the human mind.

The genius which reared monuments so stupendous and so diverse, was assuredly one of the most eminently philosophic that has ever appeared. The most striking characteristic, in fact of the talent of Aristotle, is his faculty of generalization. He observes with care, compares with sagacity, and is constantly seeking to conform isolated facts to points considered in a general view. His style, as remarkable for its clearness and precision as for the abundance of the ideas, bears upon it the impress of this habit of generalization. He never wrote anyting useless. His style is that of an elevated reason, but cold and without enthusiasm. It is simple, exact, correct, and full of information. In it judgment and experience occupy a higher stand than the imagination. Less graceful and refined than that of Plato, less elegant but equally pure with that of Theophrastus, we may regard it as the model of the philosophic style, and of the art of expressing general principles as the most complete details of the science. The destiny of Aristotle, his writings and his school are circumstances worthy of remark. Almost deified during his life, statues were raised to him, and his countrymen instituted in his honor, triumphal fetes. But soon after his death, his doctrines were altered; Theophrastus his pupil and immediate successor was almost the only one, who preserved it in all its purity. Whether other schools and other teachers, favored by their actual existence, had attracted to themselves the entire attention of their contemporaries, or, with their usual fickleness, had grown weary of the success of the peripatetic school, it is certain that the writings of the philosopher of Stagira by degrees fell into a sort of oblivion; up to the time when the Romans began to interest themselves in philosophy, the world seemed to have entirely lost sight of them, nevertheless his doctrines did not regain their proper stand till the time of the Arabs, who, in the middle ages, introduced them into Europe. From that moment they were espoused far and wide; they penetrated into all nations, they were taught in all the sciences, so much so, that during twenty centuries, in the words of Laharpe "the boundaries of the mind of Aristotle seemed to be also the boundaries of the human mind."

It was not for the mere reason that Aristotle was skilled in pharmacy or practical medicine, that we have bestowed our attention, perhaps too long, upon the life and works of this eminent man, but because, on the one hand, the extent, the variety of his knowledge and of his discoveries would not allow us to speak more briefly, and on the other, it was important, with a view to our subject, to prove that the true origin of the natural sciences may be traced back to the peripatetic school. What Aristotle achieved for zoology, Theophrastus continued for vegetables and in part for the mineral kingdom.

